# Estimation of Online State of Charge and State of Health Based on Neural Network Model Banks Using Lithium Batteries

**DOI:** 10.3390/s22155536

**Published:** 2022-07-25

**Authors:** Jong-Hyun Lee, In-Soo Lee

**Affiliations:** School of Electronic and Electrical Engineering, Kyungpook National University, Daegu 41566, Korea; whdugs8428@knu.ac.kr

**Keywords:** lithium batteries, state of charge, state of health, multilayer neural networks, long short-term memory, estimation

## Abstract

Lithium batteries are secondary batteries used as power sources in various applications, such as electric vehicles, portable devices, and energy storage devices. However, because explosions frequently occur during their operation, improving battery safety by developing battery management systems with excellent reliability and efficiency has become a recent research focus. The performance of the battery management system varies depending on the estimated accuracy of the state of charge (SOC) and state of health (SOH). Therefore, we propose a SOH and SOC estimation method for lithium–ion batteries in this study. The proposed method includes four neural network models—one is used to estimate the SOH, and the other three are configured as normal, caution, and fault neural network model banks for estimating the SOC. The experimental results demonstrate that the proposed method using the long short-term memory model outperforms its counterparts.

## 1. Introduction

Lithium batteries are secondary batteries used as power sources in various technologies, such as electric vehicles, portable devices, and energy storage devices, owing to their high-power density, low self-discharge rate, and light weight [1,2,3,4]. However, explosions caused by lithium batteries commonly result in accidents, necessitating research on highly reliable and effective battery management systems [5,6,7,8]. To improve safety, the battery management system monitors the voltage, current, and temperature of the battery. In addition, the state of charge (SOC) and state of health (SOH) are estimated through the internal parameters of the battery, which prevents a voltage imbalance between cells inside the battery pack as well as overcharge/discharge to maximize the battery performance. Therefore, accurately determining the SOC and SOH of a battery is directly related to the performance improvement of the battery management system. However, SOC and SOH cannot be measured directly and must be estimated through algorithms using the internal or external parameters of the batteries, such as voltage, current, and temperature. These parameters can be affected by the status of the operating environment and battery. Hence, modeling algorithms that reflect the particular characteristics of the battery are required. 

Several studies have been conducted on SOC and SOH estimation methods using various methods. For example, a recently proposed SOC estimation method uses a feedforward-long short-term memory model to reflect battery characteristics based on a sliding balance window [9] and an adaptive square root extended Kalman filter method that minimizes the effect of noise, regardless of the temperature [10]. Typical methods for estimating SOC and SOH include model-based, data-driven, and Coulomb counting methods [11]. Model-based methods have the advantage of being powerful and highly accurate because they are based on a deep understanding of the system. However, to develop a model that accurately describes the target system, there are practical and theoretical problems to solve. Data-driven methods are advantageous because users do not require in-depth or specific knowledge of the target system, and they can rely on the data analysis performed by the system. However, a large amount of data is required in this case. The Coulomb counting method measures the discharge current of a battery and integrates it over time to determine its current capacity. Table 1 summarizes the attributes and shortcomings of these three methods.

Recently, studies on battery estimation methods using Kalman filters have been actively conducted owing to the significant reduction in estimation error [24,25]. Some of the recently reported studies are as follows.

Rzepka et al. [26] proposed a SOC estimation method using an extended Kalman filter (EKF). In their study, to verify the performance of the proposed EKF model, they compared the EKF model tuned with the noise metric Q and the untuned EKF model. The experimental results revealed that the error of the untuned EKF model was 4%, and that of the tuned EKF was approximately 1%, indicating good performance. However, this error suggests that the EKF is sensitive to noise, and the state of the battery may change owing to degradation, thus decreasing the estimation of battery performance.

Li et al. [27] proposed a SOC and SOH estimation method using a dual-adaptive EKF. The proposed method improved the estimation performance by incorporating the estimation error of the terminal voltage and battery capacity information obtained from the equivalent circuit model into a filter with an estimation accuracy of less than 1%. However, the authors noted that the degradation was not considered, and the computational complexity should be considered when considering the degradation of the battery.

In this study, a neural network model was used to determine the degradation characteristics of the batteries. In the case of the neural network model, the relationship between the battery capacity and the parameters can be determined based on the parameters measured in the battery. The main challenge of this model is to accurately model battery aging by extracting useful characteristics from the measured signals [28].

In this study, SOH monitoring and SOC online estimation algorithms based on the SOH results are proposed. The main contributions of this study are as follows. First, the proposed method estimates the SOH of a battery using a neural network model. Second, the SOC was estimated using the estimated SOH value. Thus, the SOC and SOH parameters can be estimated simultaneously, and the proposed method can yield accurate SOC estimation results using the current state information of the battery. Finally, a neural network model bank is developed to study the degradation and nonlinear characteristics of lithium batteries.

This study offers the following improvements over the previous study [29]. First, the SOH estimation method is considerably different. In the previous study, a single discharge cycle was used to estimate the SOH, and the SOH was estimated after one discharge cycle was complete. However, this study proposes an algorithm that estimates the SOH using several battery parameters and the previous SOC. Thus, it is possible to simultaneously estimate SOC and SOH online.

Next, different parameters are used for the estimation of SOC and SOH. In the previous study, time and discharge voltage over a single cycle were used. However, the proposed method uses time, voltage, current, temperature, and the previous SOC. Moreover, this method learns the relationship between battery parameters, SOC, and SOH and yields accurate results.

Among the feasible neural network models for SOH estimation, the proposed algorithm uses a multilayer neural network (MNN) and long short-term memory (LSTM) models. The neural network model bank, which combines three models for each of the MNN and LSTM models, is used for SOC estimation. The neural network model bank consists of normal, cautious, and fault models based on learned data. The proposed algorithm selects one of the three models from the neural network model bank according to the SOH estimated by the SOH model, estimates the SOC, and simultaneously outputs the SOC and SOH results. For example, when the output of the SOH diagnostic model is normal, the SOC estimation result of the normal model of the model bank is outputted. To verify the performance of the proposed algorithm, the MNN and LSTM models were compared with NASA and Oxford battery datasets. This procedure is described in detail in Section 3.1.3.

## 2. Battery Data Analysis

This study used the battery data provided by NASA and Oxford to verify the proposed algorithm. The NASA and Oxford datasets include data from the B0005 battery and Cell 1, respectively. Using the NASA dataset, a lithium battery with a capacity of 2 Ah was discharged with a constant current of 2 A at room temperature until the battery voltage dropped to 2.7 V. Using the Oxford dataset, the 740 mAH SLPB533459H4 battery was discharged at 1 C until the battery voltage dropped to 2.7 V.

**Figure 1:**
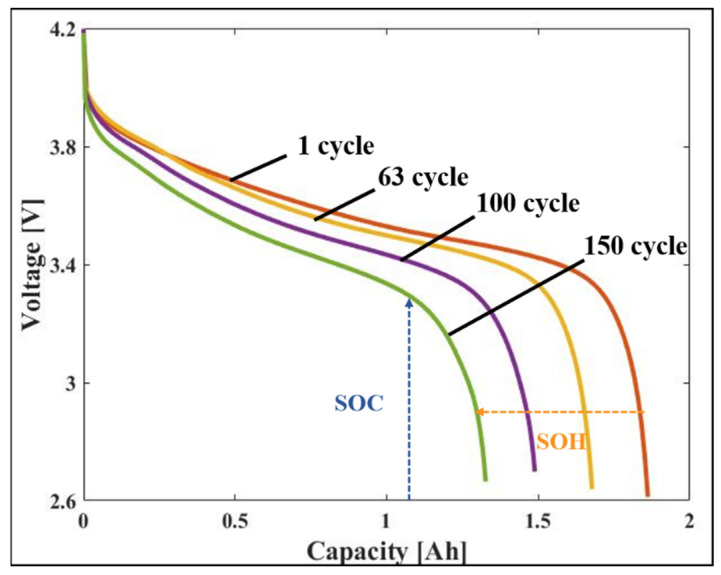
Variations of the voltage and capacity of B0005 battery.

Figure 1 shows the cycle curve of NASA’s B0005 battery. The left shift of the discharge curve indicates that the total capacity of the battery decreases and is also an indicator of the SOH. The battery voltage, which decreases with discharge time, is an indicator of the SOC.

The SOC represents the available capacity of a battery as a percentage. It is primarily used to estimate the battery availability in the battery management system. The fully charged state is indicated as 100%, and the completely discharged state is indicated as 0%. The battery capacity decreases as the degradation progresses. Thus, even if the SOC is the same, the available capacities of the new and degraded batteries differ. Therefore, an accurate estimation of the SOC is required to manage the battery efficiently [30]. The SOC is expressed as follows:

(1) $$\mathrm{SOC}=\frac{C_{Remaining}}{C_{Initial}}\times 100\;\left(\%\right),$$

where $C_{Remaining}$ is the capacity of the currently available battery, and $C_{initial}$ is the initial capacity at the time the battery is released from the factory.

The simplest method for calculating SOC is using the Coulomb counting method. However, this method cannot calculate the exact SOC value if the initial SOC setting is incorrect or if the error in the measured sensor value continues to accumulate. This study addressed these shortcomings by estimating the SOC using neural networks. 

The SOH is a performance index that represents the comparison between the initial battery and current battery capacity. It is expressed as a percentage and is 100% if the capacity of the battery is the same as the initial battery state. The SOH decreases when the battery is repeatedly used, and therefore, it can be used to estimate the remaining useful life of the battery in the battery management system [31]. The SOH is expressed as follows:

(2) $$\mathrm{SOH}=\frac{C_{Current}}{C_{Fresh}}\times 100\;\left(\%\right),\;$$

where $C_{Current}$ and $C_{Fresh}$ are the current and initial capacity of the battery, respectively. A lithium battery is considered faulty when the capacity decreases to 80% of its initial capacity [32].

In this study, the SOH is defined as a percentage reflecting the battery capacity, where the normal, caution, and fault states of the battery exhibit SOH of 90–100%, 80–90%, and <80%, respectively.

## 3. Proposed SOC and SOH Estimation Method

### 3.1. SOC Estimation Algorithm Based on the SOH Result

#### 3.1.1. Proposed SOC Estimation Based on SOH Result

**Figure 2:**
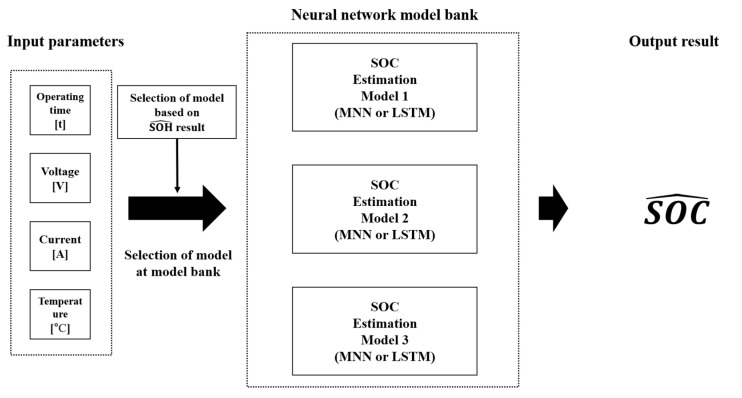
Structure of SOH estimation method.

Existing SOC estimation methods use parameters such as the voltage, current, temperature, and internal resistance to model the deterioration characteristics of a battery [33,34]. In this study, a battery SOC estimation model was developed using neural networks to determine battery characteristics. The most common neural network models are MNN and LSTM, and LSTM is a type of recurrent neural network. The characteristics of the battery change because of the degradation over time, resulting in inaccurate SOC estimation. To learn the degradation characteristics of the battery, the proposed method classifies the battery data as normal, caution, or fault according to the SOH and learns them as neural network models. Figure 2 shows the structure of the proposed SOC estimation method.

The proposed SOC estimation method involves four steps. In Step 1, the acquired battery parameters—operation time, voltage, current, and temperature—are input into the neural network model bank, and the average SOC value is calculated using the outputs. This average SOC value is used as the initial SOC value, and this step is performed only once during the algorithm execution. Step 2 involves combining the SOC values obtained in the previous step with the battery parameters and inputting them into the SOH estimation model. The SOH estimation model outputs one of three states—normal, caution, and fault—according to the estimated SOH value. In Step 3, based on the estimated SOH, the model of the current battery state is selected from the neural network model bank, and the SOC is outputted. In Step 4, the estimated SOC is used as the input parameter for the SOH estimation model.

#### 3.1.2. Structure of SOH Estimation Model

**Figure 3:**
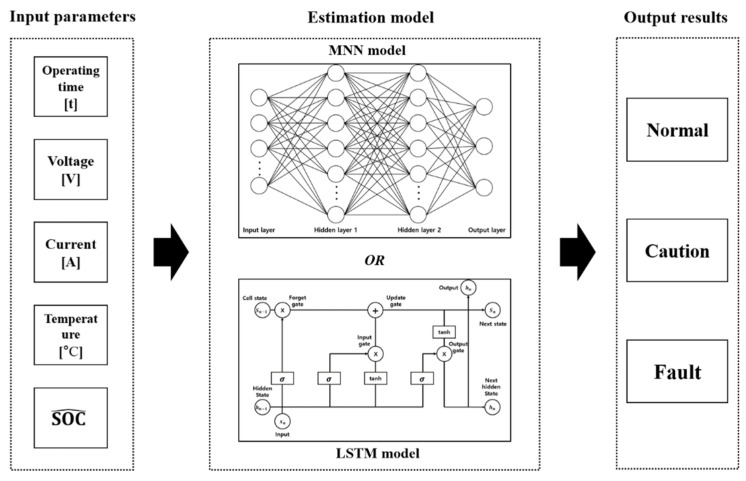
Structure of SOH estimation method.

The SOH estimation model was developed using two types of neural networks, namely MNN and LSTM. Figure 3 shows the SOH estimation model, which uses internal battery parameters such as operation time, voltage, current, temperature, and estimated SOC to estimate the SOH. 

The SOH estimation method involved the following steps. First, the battery parameters were input into the proposed SOC estimation model, and the estimated SOC was obtained. The estimated SOC, along with the current, voltage, operating time, and temperature, were combined and used as the inputs of the SOH estimation model. Thereafter, the estimated SOH was outputted from the SOH estimation model using the input parameters. If the estimated SOH was in the range of 90–100%, it was outputted as normal. By contrast, if the SOH range was 80–90%, it was outputted as caution, and when the range was <80%, it was outputted as a fault.

#### 3.1.3. SOC and SOH Online-Estimation Method

In this study, two methods were proposed—Method 1 employs the SOH estimation model without using the neural network model bank, whereas Method 2 uses the neural network model bank. Comparing the results of the two methods can help ascertain whether the neural network model bank contributes to performance improvement of the SOC and SOH.

##### Proposed Method 1: SOC Estimation without a Neural Network Model Bank

**Figure 4:**
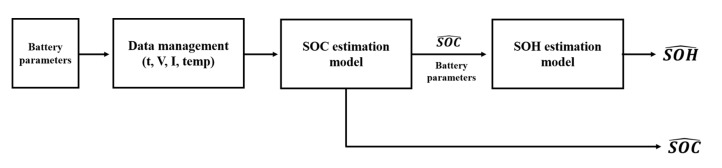
Process of proposed SOC and SOH estimation without a neural network model bank.

The process of method 1 is as follows. The battery parameter is input into the SOC estimation model without the model bank, and the SOC is estimated. The estimated SOC is then input into the SOH estimation model to estimate the SOH. Subsequently, the estimated SOC and SOH are simultaneously outputted. This process is illustrated in Figure 4.

##### Proposed Method 2: SOC Estimation with a Neural Network Model Bank

**Figure 5:**
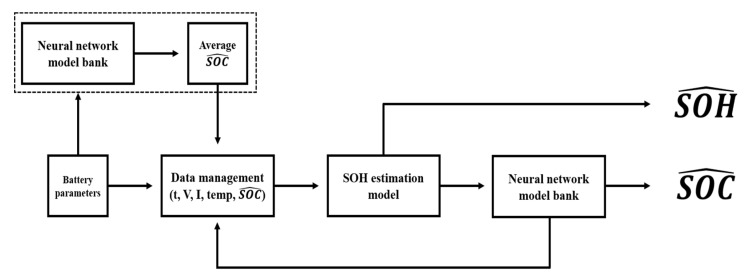
Process of proposed SOC and SOH estimation with a neural network model bank.

Figure 5 shows a schematic illustrating the process of estimating the SOC and SOH using Method 2. This process involves four steps. In Step 1, the battery parameters are input into the neural network model bank for SOC estimation, and the average SOC is calculated using the output. This average SOC is used as the initial SOC, and this step is performed only once during algorithm execution. In Step 2, the SOC obtained in Step 1 is input into the SOH estimation model along with the battery parameters. The SOH estimation model outputs one of three states—normal, caution, or fault—according to the estimated SOH. In Step 3, based on the estimated SOH, the model reflecting the current battery state is selected from the neural network model bank, and the SOC is outputted. Finally, in Step 4, the estimated SOC value is used as the input parameter of the SOH estimation model.

### 3.2. Multilayer Neural Network (MNN)

**Figure 6:**
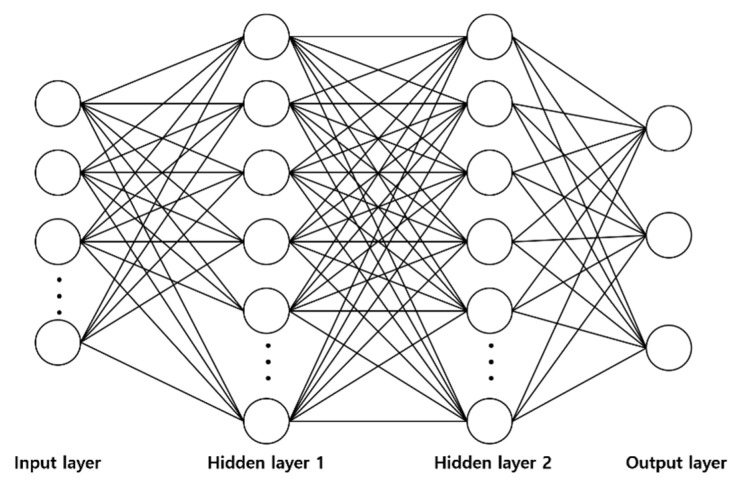
Structure of MNN.

This study used MNN and LSTM as neural network models to learn the nonlinear characteristics of lithium batteries and implement the proposed method. MNNs are widely used and contain one or more hidden layers between the input and output layers [35]. The structure of the multilayer neural network is illustrated in Figure 6.

An MNN learns via backpropagation algorithms; however, various algorithms can be used for learning. The learning algorithm propagates the error generated in the output layer to the input layer to correct the weight and minimize the error. In this study, the weights were updated using the Adam algorithm, which is a first-order gradient-based optimization algorithm for stochastic objective functions based on the adaptive estimation of low-order moments. The Adam algorithm offers the advantages of simple implementation, high computational efficiency, and suitability for problems with complex data or parameters [36]. The Adam algorithm is expressed as follows.

(3) $$m_{t}=\beta_{1}m_{t}+\left(1-\beta_{1}\right)\nabla_{\theta }f\left(\theta \right)$$

(4) $$v_{t}=\beta_{2}v_{t-1}+\left(1-\beta_{2}\right){\left(\nabla_{\theta }f\left(\theta \right)\right)}^{2}$$

Because m and v have initial values of 0, a bias close to 0 is expected at the start of learning, making them unbiased.

(5) $$\hat{m_{t}}=\frac{m_{t}}{1-\beta_{1}^{t}},$$

(6) $$\hat{v_{t}}=\frac{v_{t}}{1-\beta_{2}^{t}\;},\;$$

(7) $$\theta =\theta -\frac{\eta }{\sqrt{\hat{v_{t}}+\epsilon }}\hat{m_{t}},$$

where $m_{t}$ is the initial 1st moment vector, $v_{t}$ is the initial 2nd moment vector, $\beta_{1}$ and $\beta_{2}$ are exponential decay rates for the moment estimates, $\beta_{1}$ is 0.9, $\beta_{2}$ is 0.999, t is the time-step initialization, θ is the initial parameter vector, $\nabla_{\theta }f\left(\theta \right)$ is the stochastic objective function with parameters θ, and ϵ is $10^{-8}$.

### 3.3. Long Short-Term Memory (LSTM)

LSTM is a model that solves the long-term dependencies of the existing recurrent neural network. It enables storing both long- and short-term information by adding a cell state and a gate to the existing neural network model. The LSTM model consists of a cell state, forget, input, and output gate. LSTM is expressed as follows [37].

Step 1. Forget gate

(8) $$f_{t}=\sigma \left(W_{f}\cdot \left[h_{t-1},x_{t}\right]+b_{f}\right)$$

Step 2. Input gate

(9) $$i_{t}=\sigma \left(W_{i}\cdot \left[h_{t-1},x_{t}\right]+b_{i}\right)$$

(10) $${\tilde{C}}_{t}=tanh\left(W_{C}\cdot \left[h_{t-1},x_{t}\right]+b_{C}\right)$$

Step 3. Cell-state update

(11) $$C_{t}=f_{t}\cdot C_{t-1}+i_{t}\cdot {\tilde{C}}_{t}$$

**Figure 7:**
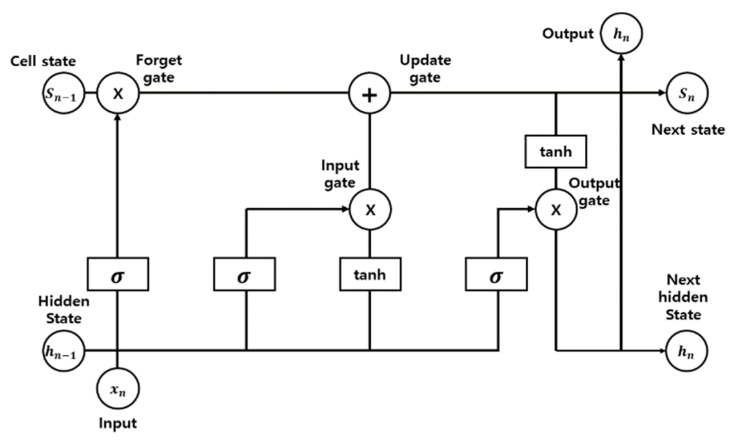
Structure of an LSTM.

Step 4. Output gate

(12) $$o_{t}=\sigma \left(W_{o}\cdot \left[h_{t-1},x_{t}\right]+b_{o}\right)$$

(13) $$h_{t}=o_{t}\cdot \tanh\left(C_{t}\right)$$

where, $h_{t-1}$ is the past parameter, $x_{t}$ is the current input parameter, W  is the weight, b  is the bias, $f_{t}$ is the value of the forget gate, and $i_{t}$ and ${\tilde{C}}_{t}$ are the values calculated using the sigmoid function and activation function, respectively. $C_{t}$ is the value updated in the cell state, $o_{t}$ is the value of the output gate, and $h_{t}$ is the output. The structure of LSTM is shown in Figure 7.

## 4. Experiment and Results

In this study, the model was trained using the NASA and Oxford battery datasets, and the performance of the proposed method was verified. The computational setup used for training was Ryzen 5600X, RTX 3070, and 16 GB of RAM, and training was conducted using Python 3.6, TensorFlow (version 2.2), and the Keras library.

To verify the proposed method, the SOC and SOH models used MNN and LSTM. The structure of the SOC estimation model using the MNN was 4-256-128-64-1. The Adam algorithm was used for learning, the rectified linear unit function was used as an activation function for each hidden layer, and the number of epochs was set to 20,000. The structure of the SOC estimation model using LSTM was 4-128-64-32-1. The Adam algorithm was used as a learning algorithm, the sigmoid function was used as an activation function for each LSTM layer, and the number of epochs was set to 5000. 

In the case of the SOH estimation model, each of the MNN and LSTM models had the same structure as that in the SOC estimation model. The learning algorithm and activation function of the hidden layer were identical to those used in the SOC estimation model, and the epochs were set to 10,000 and 3000 for the MNN and LSTM models, respectively.

The learning data consisted of the NASA and Oxford battery datasets. In the former case, the B0005 battery dataset was used, along with 6 cycles for testing and 144 cycles for learning. The test data were used when the SOH was 95, 90, 85, 80, 77, and 75, which corresponded to cycles 41, 63, 78, 100, 125, and 150, respectively. In the case of learning, to configure the neural network model bank, the battery cycle data were learned in the normal, caution, and fault model according to the SOH. The normal model learned in 1–62 cycles, the caution model in 64–99 cycles, and the fault model in 101–149 cycles. The SOH estimation model was trained over 144 cycles. 

In the case of the Oxford dataset, only Cell 2 battery data were used, and a total of 6 cycles were used for testing and 71 for learning. The test data were used when the SOH values were 97, 93, 87, 83, 77, and 75, which corresponded to cycles 6, 15, 28, 42, 70, and 78 in the dataset. For learning, the normal model was trained in 1–20 cycles, the caution model in 21–53 cycles, and the fault model in 54–77 cycles. The SOH estimation model was trained over 71 cycles.

The performance of the proposed SOC and SOH estimation methods using the NASA battery dataset are presented in Table 2 and Table 3, which show the SOC and SOH estimation results, respectively. The error was calculated using the mean absolute error (MAE), expressed as follows:

(14) $$MAE=\frac{1}{n}\overset{n}{\underset{i=1}{\sum }}\left|y_{i}-\hat{y}\right|,$$

where n is the total number of parameters, $y_{i}$ is the target value, and $\hat{y}$ is the estimated value.

By employing Method 1, the SOC estimation results using LSTM and MNN had average errors of 0.094 and 0.124, respectively; using Method 2, the average errors were 0.091 and 0.294, respectively. When Method 2 using LSTM estimated 63 cycles, the estimation result showed a higher error than the other models, but other cycles showed a lower error than the other models. Therefore, the average error was lower than that of the other models. Thus, this model offered the best performance.

**Figure 8:**
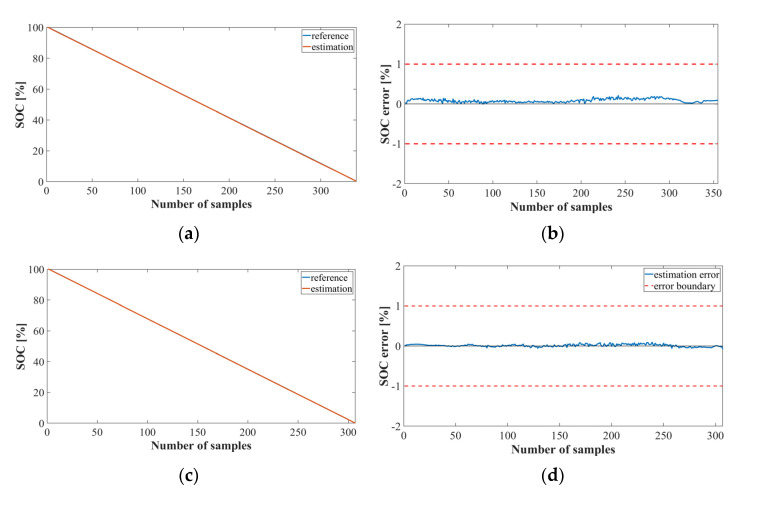

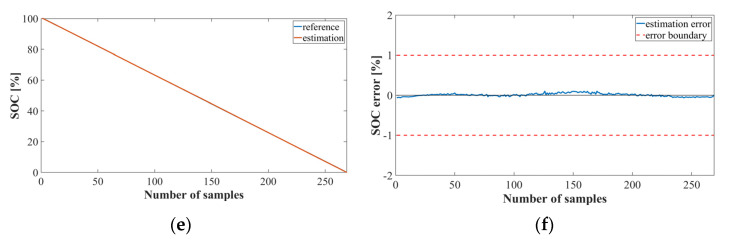
SOC estimation results for Method 2 using the LSTM (NASA dataset): (**a**) SOC estimation result; (**b**) error at cycle 41; (**c**) SOC estimation result; (**d**) error at cycle 78; (**e**) SOC estimation result; (**f**) error at cycle 125.

Figure 8 shows the graph of the SOC estimation result obtained by implementing Method 2 using LSTM, which represents the results of 41, 78, and 125 cycles of the NASA datasets. Figure 8a,c,e show the SOC estimation results for each cycle, and Figure 8b,d,f show the SOC estimation error. Evidently, the performance of the bank model using the LSTM was accurately estimated with an average error of approximately 0.

**Figure 9:**
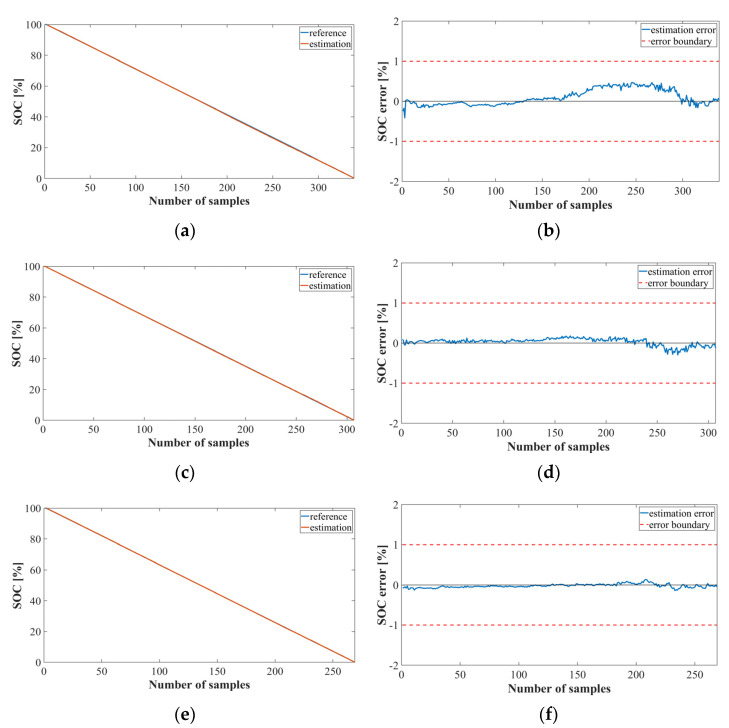
SOC estimation results for Method 2 using the MNN (NASA dataset): (**a**) SOC estimation result; (**b**) error at cycle 41; (**c**) SOC estimation result; (**d**) error at cycle 78; (**e**) SOC estimation result; (**f**) error at cycle 125.

Figure 9 shows a graph of the SOC estimation results obtained by implementing Method 2 using the MNN. Even in the bank model using the MNN, the error range is less than +1 and −1. However, as the estimation proceeds, the error becomes larger than that of the LSTM model.

Table 3 lists the errors of the SOH estimation models derived from Method 2 using LSTM and MNN with the NASA dataset. The average errors for the SOH estimation were 0.734 and 1.332 using LSTM and MNN, respectively. It was confirmed that using LSTM is better than MNN for Method 2 because the estimation results of cycle numbers beyond 125 were accurate.

**Figure 10:**
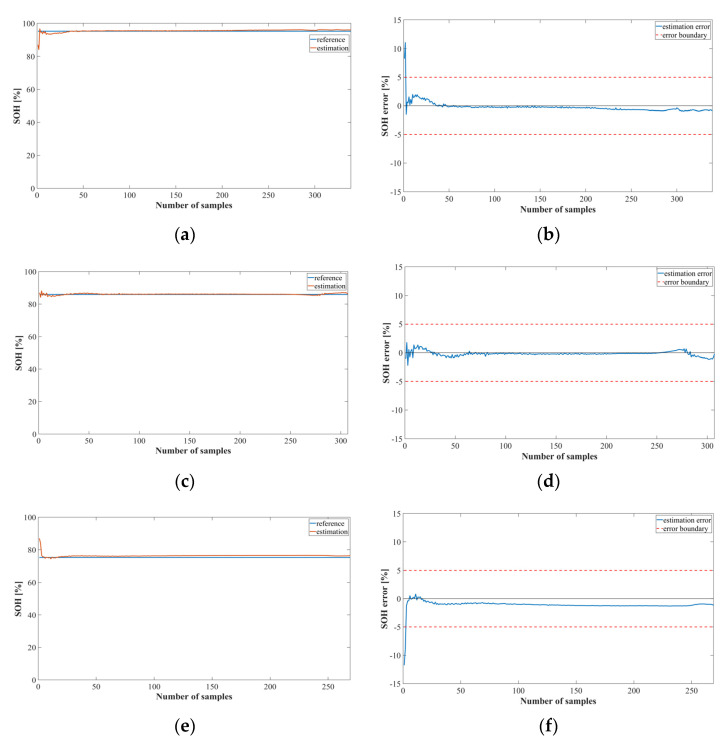
SOH estimation results for Method 2 using the LSTM (NASA dataset): (**a**) SOH estimation result; (**b**) error at cycle 41; (**c**) SOH estimation result; (**d**) error at cycle 78; (**e**) SOH estimation result; (**f**) error at cycle 125.

**Figure 11:**
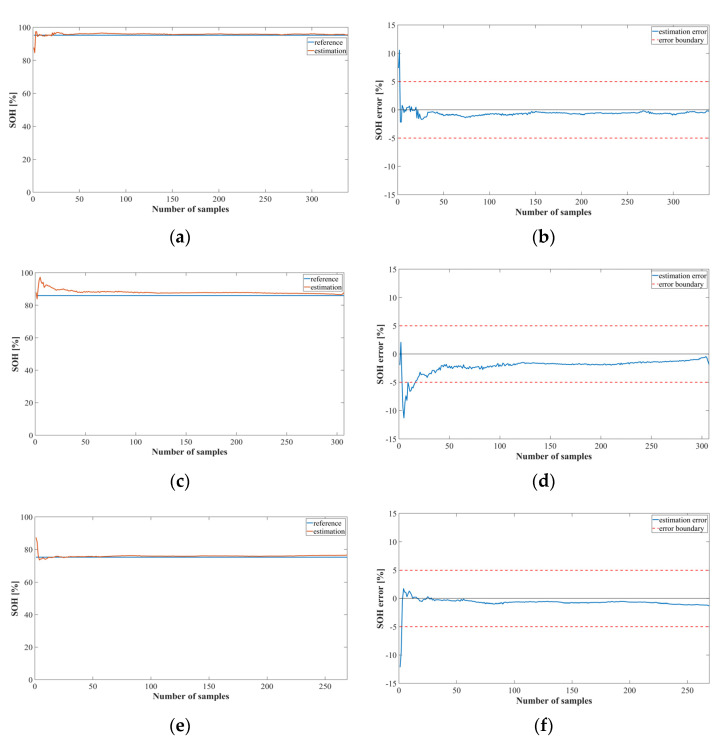
SOH estimation results for Method 2 using the MNN (NASA dataset): (**a**) SOH estimation result; (**b**) error at cycle 41; (**c**) SOH estimation result; (**d**) error at cycle 78; (**e**) SOH estimation result; (**f**) error at cycle 125.

Figure 10 and Figure 11 show the output of the SOH estimation model using the LSTM and MNN, respectively. The test datasets were identical to those used in the SOC estimation experiment. Figure 10a,c,e show the SOH estimation results for each cycle, and Figure 10b,d,f show the corresponding SOH estimation errors.

The SOH estimation results were reasonably accurate, except for the first part, which exhibited a large error. At the onset of battery operation, the values were similar for the normal, caution, and fault states, and the average of the learned SOH value range was the output. However, this error did not significantly affect the SOC estimation result because the SOC model estimated the first part of the normal, caution, and fault models as essentially the same value.

Figure 11 shows the results of the SOH estimation model using the MNN. The SOH estimation model using the MNN performed similarly to the LSTM model at cycles 41 and 125. However, at cycle 78, the estimation error was greater than that of the LSTM model.

The performance of the proposed SOC and SOH estimation methods using the Oxford battery dataset are listed in Table 4 and Table 5, respectively.

As is evident from Table 4, the results of Method 1 using LSTM and MNN exhibited average errors of 0.245 and 0.583, respectively, whereas the corresponding average errors for Method 2 using the LSTM and MNN were 0.218 and 0.306, respectively. Although the difference in the estimation error between Method 2 and Method 1 using the LSTM model was not very large, the average error was lower than that of the non-model bank employing LSTM. Moreover, Method 2, using LSTM, yielded better results than the non-model-bank model using LSTM.

**Figure 12:**
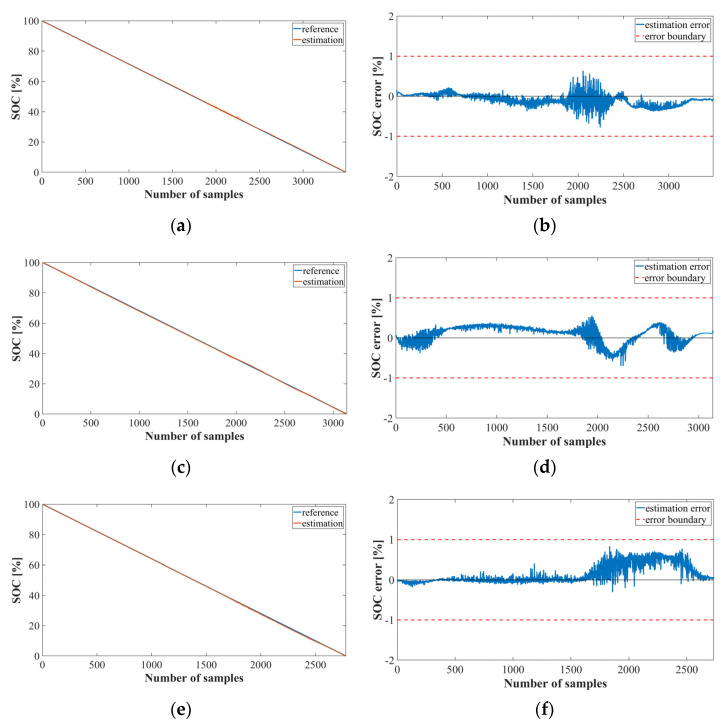
SOC estimation results for Method 2 using the LSTM (Oxford dataset): (**a**) SOC estimation result; (**b**) error at cycle 6; (**c**) SOC estimation result; (**d**) error at cycle 28; (**e**) SOC estimation result; (**f**) error at cycle 70.

Figure 12 shows a graph of the SOC estimation result obtained by implementing Method 2 using the LSTM and Oxford datasets for cycles 6, 28, and 70. The error of the bank model using LSTM was estimated to be less than +1 and −1.

**Figure 13:**
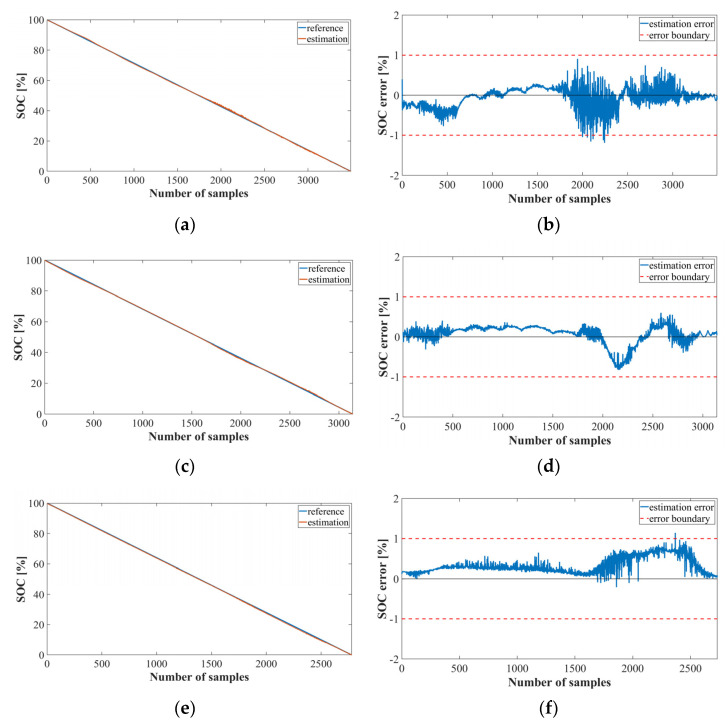
SOC estimation results for Method 2 using the MNN (Oxford dataset): (**a**) SOC estimation result; (**b**) error at cycle 6; (**c**) SOC estimation; (**d**) error at cycle 28; (**e**) SOC estimation; (**f**) error at cycle 70.

Figure 13 shows a graph of the SOC estimation results obtained by implementing the bank model using MNN. It was confirmed that Method 2 using MNN increased the error significantly after the 2000 sample than Method 2 using LSTM, and the average error exceeded +1 and −1. Thus, the model using LSTM performed better than the model using MNN.

Table 5 shows the estimation errors of the SOH estimation models implemented using the LSTM and MNN with the Oxford datasets, which had average errors of 1.071 and 1.86, respectively. It was confirmed that the SOH estimation model using LSTM was superior to the MNN because the estimation result for the remaining cycles, excluding the first 28 cycles, was very good.

**Figure 14:**
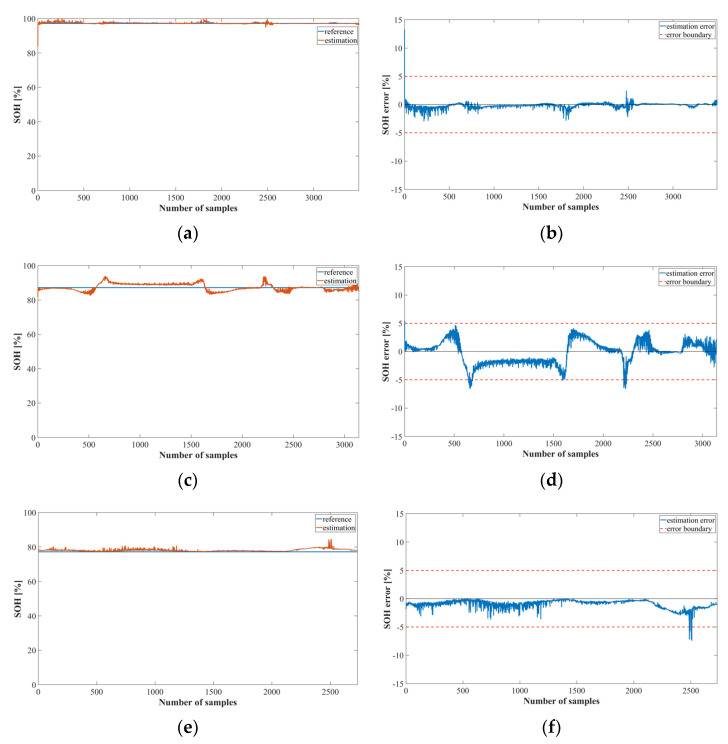
SOH estimation results for Method 2 using the LSTM (Oxford dataset): (**a**) SOH estimation result; (**b**) error at cycle 6; (**c**) SOH estimation result; (**d**) error at cycle 28; (**e**) SOH estimation result; (**f**) error at cycle 70.

Figure 14 shows the estimation results of the SOH estimation model using LSTM. It can be observed that the results for cycles 28 and 70 have a greater error than those for cycle 6. Because Oxford’s battery parameters have very similar values in a normal and caution state, the error increases in the corresponding cycle for both the LSTM and the MNN models.

**Figure 15:**
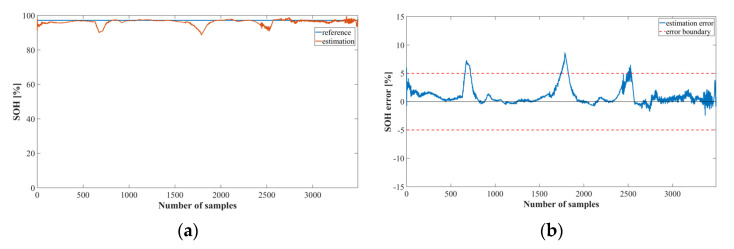

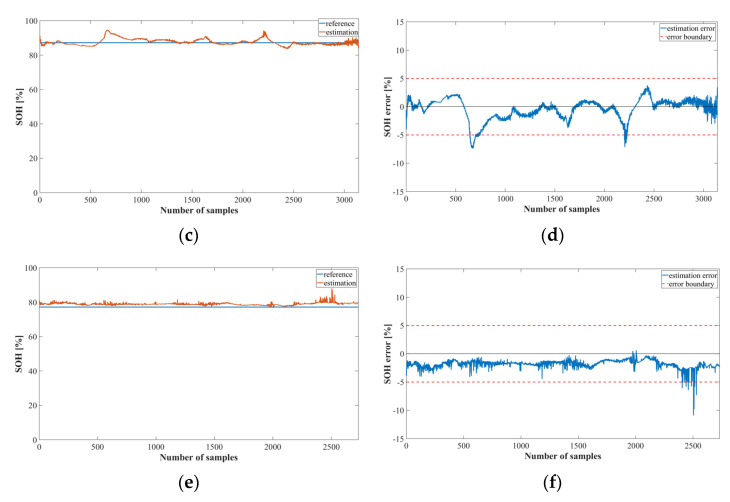
SOH estimation results using the proposed Method 2 with MNN and the Oxford dataset: (**a**) SOH estimation result at 6 cycles; (**b**) SOH estimation error at 6 cycles; (**c**) SOH estimation result at 28 cycles; (**d**) SOH estimation error at 28 cycles; (**e**) SOH estimation result at 70 cycles; (**f**) SOH estimation error at 70 cycles.

Figure 15 shows the graph of the SOC estimation result obtained by implementing proposed method 2 using the MNN model. The overall trend is similar to that of the LSTM model, but the error was larger than that of the LSTM model. Thus, it was confirmed that the Oxford data also estimated the proposed method using LSTM better than the MNN.

Table 2 and Table 4 list the errors in the SOC estimation results. Method 1 does not use a neural network model bank, whereas Method 2 uses a neural network model bank. The estimation results of Methods 1 and 2 using LSTM listed in Table 2 and Table 4, respectively, show similar trends in each cycle. However, the size of the error is different. The estimation results of Methods 1 and 2 using MNN exhibited an increase in error as the number of cycles increased. The characteristics changed suddenly owing to the degradation of the battery; therefore, learning these characteristics accurately in MNN was not possible. In the case of LSTM, the nonlinear characteristics of the battery were learned more accurately than in MNN, with an algorithm that receives the data from the previous cycle as the input for the succeeding cycle.

Table 3 and Table 5 list the errors in SOH estimation. The SOH estimation yields a larger error than that of the SOC estimation result. This is because the change in the battery parameters according to the change in the SOH is not large, resulting in an incorrect estimation of the SOH. In particular, this problem is apparent between the caution and fault data groups, as observed in Table 3. The error increases at 100 cycles and then decreases at 150 cycles when the battery is severely degraded. To reduce this error, this study used an LSTM model, specifically designed for time-series data learning, and the results show that the SOH estimation is reasonably accurate compared to that obtained using the model with MNN.

## 5. Conclusions

In this study, we proposed an online state of charge (SOC) and state of health (SOH) estimation method based on a neural network model bank comprising four neural networks (NNs)—one was used for SOH estimation using either a multilayer neural network (MNN) or long short-term memory (LSTM), whereas the remaining three were configured as neural network model banks, and the SOC was estimated using MNN and LSTM. The three neural network model banks were labeled Model 1, Model 2, and Model 3 according to the learned data from the normal, caution, and fault data groups of the battery datasets, respectively. Subsequently, one of the three neural network models was selected according to the results of the SOH estimation model, and the SOC estimation result was outputted.

The experiment used MNN and LSTM to verify the performance of the proposed model. In addition, we confirmed the effectiveness of the four methods using the model-bank models with MNN and LSTM and compared them with the corresponding non-model-bank models with MNN and LSTM.

For the NASA dataset, we confirmed that the average errors of the SOC estimation of the model-bank models with LSTM and MNN were 0.091 and 0.294, respectively, and the non-model-bank models with LSTM and MNN were 0.094 and 0.124, respectively, indicating that LSTM resulted in a better performance in both cases. The LSTM and MNN models had average errors of 0.734 and 1.332, respectively.

For the Oxford dataset, we confirmed that the average errors of the SOC estimation of the model-bank models with LSTM and MNN were 0.218 and 0.306, respectively, and those for the LSTM and MNN non-model-bank models were 0.245 and 0.583, respectively, indicating that the LSTM performed better. The LSTM and MNN models had average errors of 1.071 and 1.86, respectively.

Therefore, in terms of SOC estimation, the model using LSTM offered good estimation performance in all tests, and the model using MNN yielded higher errors for the caution and fault state data groups. The results indicated that LSTM learned the change in characteristics due to battery degradation better than MNN. In addition, the SOH estimation results revealed that the model using LSTM was more accurate than that using MNN.

In future research, we plan to apply the proposed method in a real environment to evaluate its practical utility.

## Figures and Tables

**Table 1:** Advantages and disadvantages of model-based, data-driven, and coulomb-counting methods.

Method	Advantages	Disadvantages	Example Model
Model-based [12,13]	▪ Reliable and accurate ▪ Universal validity	▪ Requires extensive domain knowledge ▪ Long development time	▪ Equivalent circuit model Electrochemical model ▪ Kalman filter
Data-driven [14,15,16,17,18,19,20,21]	▪ Short development time ▪ Does not require much specialized knowledge	▪ Requires a large amount of data	▪ Neural network ▪ Deep learning ▪ Look-up table
Coulomb counting [22,23]	▪ Simple to implement	▪ Errors accumulate over time	▪ Coulomb counting

**Table 2:** SOC estimation error for each model using the NASA battery dataset.

	Cycle 41	Cycle 63	Cycle 78	Cycle 100	Cycle 125	Cycle 150	Average
Method 1 (LSTM)	0.108	0.079	0.088	0.055	0.058	0.181	0.094
Method 1 (MNN)	0.173	0.068	0.196	0.121	0.06	0.129	0.124
Method 2 (LSTM)	0.083	0.216	0.025	0.049	0.031	0.142	0.091
Method 2 (MNN)	0.199	0.115	0.158	0.123	0.226	0.944	0.294

**Table 3:** SOH estimation error for each model using the NASA battery dataset.

	Cycle 41	Cycle 63	Cycle 78	Cycle 100	Cycle 125	Cycle 150	Average
Method 2 (LSTM)	0.558	0.565	0.328	0.957	1.082	0.914	0.734
Method 2 (MNN)	0.719	0.808	1.995	2.428	0.795	1.252	1.332

**Table 4:** SOC estimation error for each model using the Oxford battery dataset.

	Cycle 6	Cycle 15	Cycle 28	Cycle 42	Cycle 70	Cycle 78	Average
Method 1 (LSTM)	0.138	0.179	0.296	0.153	0.287	0.417	0.245
Method 1 (MNN)	0.621	0.418	0.433	0.516	0.61	0.902	0.583
Method 2 (LSTM)	0.132	0.117	0.196	0.205	0.27	0.392	0.218
Method 2 (MNN)	0.244	0.276	0.297	0.271	0.32	0.431	0.306

**Table 5:** SOH estimation error for each model using the Oxford battery dataset.

	Cycle 6	Cycle 15	Cycle 28	Cycle 42	Cycle 70	Cycle 78	Average
Method 2 (LSTM)	0.296	0.929	1.6	1.399	0.933.	1.272	1.071
Method 2 (MNN)	1.141	1.053	1.353	2.396	1.782	3.439	1.86

## Data Availability

Publicly available datasets were analyzed in this study. This data can be found here: [https://ti.arc.nasa.gov/tech/dash/groups/pcoe/prognostic-data-repository/] (accessed on 22 December 2021). and [https://ora.ox.ac.uk/objects/uuid:03ba4b01-cfed-46d3-9b1a-7d4a7bdf6fac] (accessed on 22 December 2021).

## References

[1] Gao Z., Chin C.S., Chiew J.H.K., Jia J., Zhang C. (2017). Design and Implementation of a Smart Lithium-Ion Battery System with Real-Time Fault Diagnosis Capability for Electric Vehicles. Energies.

[2] Cho T.H., Hwang H.R., Lee J.H., Lee I.S. (2018). Comparison of Intelligent Methods of SOC Estimation for Battery of Photovoltaic System. Int. J. Adv. Comput. Sci. Appl..

[3] Park J.H., Lee J.H., Kim S.J., Lee I.S. (2020). Real-Time State of Charge Estimation for Each Cell of Lithium Battery Pack Using Neural Networks. Appl. Sci..

[4] Seh Z.W., Sun Y., Zhang Q., Cui Y. (2016). Designing high-energy lithium–sulfur batteries. Chem. Soc. Rev..

[5] Xiong R., Li L., Tian J. (2018). Towards a smarter battery management system: A critical review on battery state of health monitoring methods. J. Power Sources.

[6] Shen M., Gae Q. (2019). A review on battery management system from the modeling efforts to its multiapplication and integration. Int. J. Energy Res..

[7] Zhao Y., Liu P., Wang Z., Zhang L., Hong J. (2017). Fault and defect diagnosis of battery for electric vehicles based on big data analysis methods. Appl. Energy.

[8] Chen Z., Xiong R., Tian J., Shang X., Lu J. (2016). Model-based fault diagnosis approach on external short circuit of lithium-ion battery used in electric vehicles. Appl. Energy.

[9] Wang S., Takyi-Aninakwa P., Jin S., Yu C., Fernandez C., Stroe D.I. (2022). An improved feedforward-long short-term memory modeling method for the whole-life-cycle state of charge prediction of lithium-ion batteries considering current-voltage-temperature variation. Energy.

[10] Jiang C., Wang S., Wu B., Fernandez C., Xiong X., Coffie-Ken J. (2021). A state-of-charge estimation method of the power lithium-ion battery in complex conditions based on adaptive square root extended Kalman filter. Energy.

[11] How D.N., Hannan M.A., Lipu M.H., Ker P.J. (2019). State of charge estimation for lithium-ion batteries using model-based and data-driven methods: A review. IEEE Access.

[12] Corno M., Bhatt N., Savaresi S.M., Verhaegen M. (2014). Electrochemical model-based state of charge estimation for Li-ion cells. IEEE Trans. Control Syst. Technol..

[13] He H., Xiong R., Fan J. (2011). Evaluation of lithium-ion battery equivalent circuit models for state of charge estimation by an experimental approach. Energies.

[14] Sun F., Hu X., Zou Y., Li S. (2011). Adaptive unscented Kalman filtering for state of charge estimation of a lithium-ion battery for electric vehicles. Energy.

[15] Wu B., Han S., Shin K.G., Lu W. (2018). Application of artificial neural networks in design of lithium-ion batteries. J. Power Sources.

[16] Chemali E., Kollmeyer P.J., Preindl M., Ahmed R., Emadi A. (2017). Long short-term memory networks for accurate state-of-charge estimation of Li-ion batteries. IEEE Trans. Ind. Electron..

[17] Shen P., Ouyang M., Lu L., Li J., Feng X. (2017). The co-estimation of state of charge, state of health, and state of function for lithium-ion batteries in electric vehicles. IEEE Trans. Veh. Technol..

[18] Barai A., Widanage W.D., Marco J., McGordon A., Jennings P. (2015). A study of the open circuit voltage characterization technique and hysteresis assessment of lithium-ion cells. J. Power Sources.

[19] Ng M.F., Zhao J., Yan Q., Conduit G.J., Seh Z.W. (2020). Predicting the state of charge and health of batteries using data-driven machine learning. Nat. Mach. Intell..

[20] Severson K.A., Attia P.M., Jin N., Perkins N., Jiang B., Yang Z., Chen M.H., Aykol M., Herring P.K., Fraggedakis D. (2019). Data-driven prediction of battery cycle life before capacity degradation. Nat. Energy.

[21] Attia P.M., Grover A., Jin N., Severson K.A., Markov T.M., Liao Y.-H., Chen M.H., Cheong B., Perkins N., Yang Z. (2020). Closed-loop optimization of fast-charging protocols for batteries with machine learning. Nature.

[22] Ng K.S., Moo C.S., Chen Y.P., Hsieh Y.C. (2009). Enhanced coulomb counting method for estimating state-of-charge and state-of-health of lithium-ion batteries. Appl. Energy.

[23] Truchot C., Dubarry M., Liaw B.Y. (2014). State-of-charge estimation and uncertainty for lithium-ion battery string. Appl. Energy.

[24] Jang K.-W., Chung G.-B. (2012). A SOC Estimation using Kalman Filter for Lithium-Polymer Battery. Trans. Korean Inst. Power Electron..

[25] Xiong R., Sun F., Chen Z., He H. (2014). A data-driven multi-scale extended Kalman filtering based parameter and state estimation approach of lithium-ion polymer battery in electric vehicles. Appl. Energy.

[26] Rzepka B., Bischof S., Blank T. (2021). Implementing an Extended Kalman Filter for SoC Estimation of a Li-Ion Battery with Hysteresis: A Step-by-Step Guide. Energies.

[27] Li J., Ye M., Gao K., Xu X., Wei M., Jiao S. (2021). Joint estimation of state of charge and state of health for lithium-ion battery based on dual adaptive extended Kalman filter. Int. J. Energy Res..

[28] Chemali E., Kollmeyer P.J., Preindl M., Emadi A. (2018). State-of-charge estimation of Li-ion batteries using deep neural networks: A machine learning approach. J. Power Sources.

[29] Lee J.H., Lee I.S. (2021). Lithium battery SOH monitoring and an SOC estimation algorithm based on the SOH result. Energies.

[30] Zhang D., Dey S., Perez H.E., Moura S.J. Remaining useful life estimation of lithium-ion batteries based on thermal dynamics. Proceedings of the 2017 American Control Conference (ACC).

[31] El-Dalahmeh M., Al-Greer M., El-Dalahmeh M.A., Short M. (2020). Time-Frequency Image Analysis and Transfer Learning for Capacity Prediction of Lithium-Ion Batteries. Energies.

[32] Tang X., Wang Y., Zou C., Yao K., Xia Y., Gao F. (2019). A novel framework for Lithium-ion battery modeling considering uncertainties of temperature and aging. Energy Convers. Manag..

[33] Wang D., Bao Y., Shi J. (2017). Online lithium-ion battery internal resistance measurement application in state-of-charge estimation using the extended Kalman filter. Energies.

[34] Song C., Shao Y., Song S., Peng S., Zhou F., Chang C., Wang D. (2017). Insulation resistance monitoring algorithm for battery pack in electric vehicle based on extended Kalman filtering. Energies.

[35] Hannan M.A., Lipu M.S.H., Hussain A., Saad M.H., Ayob A. (2018). Neural network approach for estimating state of charge of lithium-ion battery using backtracking search algorithm. IEEE Access.

[36] Kingma D.P., Ba J. (2014). Adam: A method for stochastic optimization. arXiv.

[37] Chang Z., Zhang Y., Chen W. (2019). Electricity price prediction based on hybrid model of adam optimized LSTM neural network and wavelet transform. Energy.

